# The effect of growth phase on the surface properties of three oleaginous microalgae (*Botryococcus* sp. FACGB-762, *Chlorella* sp. XJ-445 and *Desmodesmus bijugatus* XJ-231)

**DOI:** 10.1371/journal.pone.0186434

**Published:** 2017-10-18

**Authors:** Ling Xia, Rong Huang, Yinta Li, Shaoxian Song

**Affiliations:** 1 Hubei Key Laboratory of Mineral Resources Processing and Environment, Wuhan, Hubei, China; 2 School of Resources and Environmental Engineering, Wuhan University of Technology, Wuhan, Hubei, China; 3 Doctorado Institucional de Ingeniería y Ciencia de Materiales, Universidad Autonoma de San Luis Potosi, San Luis Potosi, C.P., Mexico; National Research Council of Italy, ITALY

## Abstract

The effects of growth phase on the lipid content and surface properties of oleaginous microalgae *Botryococcus* sp. FACGB-762, *Chlorella* sp. XJ-445 and *Desmodesmus bijugatus* XJ-231 were investigated in this study. The results showed that throughout the growth phases, the lipid content of microalgae increased. The surface properties like particle size, the degree of hydrophobicity, and the total concentration of functional groups increased while net surface zeta potential decreased. The results suggested that the growth stage had significant influence not only on the lipid content but also on the surface characteristics. Moreover, the lipid content was significantly positively related to the concentration of hydroxyl functional groups in spite of algal strains or growth phases. These results provided a basis for further studies on the refinery process using oleaginous microalgae for biofuel production.

## Introduction

Oil-accumulating microalgae are regarding as promising feedstocks for biodiesel production and have drawn a lot of attentions in recent decades [1]. Nevertheless, microalgae-based biofuel production has not yet been commercialized to large-scale. It is mainly due to the high-energy inputs and cost constraints associated with the cultivation and harvesting of microalgal cells [2]. However, the efficiencies of both mass cultivation and harvesting of microalgal cells are much influenced by the physico-chemical surface properties [3, 4].

On the cultivation side, to improve microalgal biomass productivity in photo-bioreactors (PBRs), new strategies should be adopted to avoid biofouling, which is caused by the attachment of cells to the walls of PBRs. Biofilm would lower biomass productivity by preventing effective light penetration into the suspension, and making algal biomass attach to PBR walls inaccessible for harvesting [3, 5]. On the other hand, microalgal biofilm formed on the surface of the material allows an attached cultivation mode instead of suspended cultivation of microalgae for metabolite production, which has gained many attentions in recent years [5, 6]. For both promotion and controlling microalgal cultivation, knowledge about surface physicochemical properties is essential [6, 7].

Regarding the algal biomass harvesting process, currently applied methods include chemical coagulation/flocculation, gravity sedimentation, flotation, filtration, centrifugation and electrical-based processes. No matter which method is used, algal surface properties had a profound impact on solid-liquid separation of algal suspension [4]. Based on the studies of algal biomass separation in wastewater treatment processes, cellular surface area was first identified as a useful preliminary indicator for coagulant dosage addition [4]. Later, Henderson et al. (2010) reported a stronger correlation between the coagulant dosage and the charge density of algal cells [8]. Also, the microalgal hydrophobicity played a significant role in the algal flotation performance [9], and the ratio of coagulant dosage to surface functional group concentration determined the harvesting efficiency [10]. Therefore, a better knowledge of microalgal cell surface will help for the determination of harvesting technology, especially for the low value by-product biofuel production.

Moreover, it was reported that the growth phase of algal cells can influence on the efficiency of biomass harvesting associated with settling, tangential flow filtration [11], flocculation [12], and flotation [10]. In addition, it was well stated that the algal properties such as cell size, mobility, surface charge and concentration of functional groups, and so on, had significant impact on the solid-liquid separation of alga [4, 13]. However, there is currently little information available on the possible effects of growth phase on the surface properties of microalgae prior to this study.

The objectives of this research were to understand influence of the growth phase on the surface characteristics of the oleaginous microalgae *Botryococcus* sp. FACGB-762, *Chlorella* sp. XJ-445 and *Desmodesmus bijugatus* XJ-231. Cellular surface properties of the three microalgae at different growth phases were characterized in regard to surface charge, concentration of functional groups, sample size distribution, hydrophobicity and surface free energy. The relationships between lipid content and the characterized cell surface properties were also analyzed.

## Materials and methods

### Organisms and culture

The organism *Botryococcus* sp. FACGB-762 was obtained from freshwater algae culture collection of the Institute of Hydrobiology (FACHB-Collection) of the Chinese Academy of Sciences (Hubei, China). *Chlorella* sp. XJ-445 and *Desmodesmus bijugatus* XJ-231 were gifted by prof. Chunxiang Hu of Institute of Hydrobiology, Chinese Academy of Sciences, China. They were both isolated from northern Xinjiang province of China and identified by classical morphological methods. Stock cultures were grown in BG-11 medium with sterile distilled water [14].

For experiments, cultures were grown in 500 mL Erlenmeyer flasks filled with 350 mL of BG-11 medium and aerated with continuous sterile filtered air. The experiments were conducted in triplicate and used axenic cultures during the whole period. The cultures were performed at room temperature (25±1°C) with a 14/10h light-dark cycle at a photosynthetically active photon flux density (PPFD) of approximately 100 μmol photons m^−2^ s^−1^ using white LEDs as light sources in a temperature-controlled incubator. The microalgal biomass was harvested at different growth phases and used for the subsequent analysis.

### Analysis of biomass and lipid content

The dry weight of the algal biomass was determined gravimetrically, and biomass concentration was expressed in terms of dry weight. The samples were harvested by centrifugation. The pellets were then washed three times with ultrapure water, freeze-dried, and weighed. Total lipid content of the cells was determined by the modified method of Bligh and Dyer (1959) using 1:2 chloroform: methanol (v/v) [15].

### Surface physiochemical properties

#### Cell size and surface charge measurements

The hydrodynamic size of algal cells at different growth phase was determined by a Malvern HYDRO 2000 MU Laser particle size analyzer (UK) at 25°C. The surface charge was measured by a zeta potential analyzer (Malvern, Zetasizer Nano, ZS 90) at 25°C [16].

#### Surface contact angle measurements

Contact angle was measured with the sessile drop technique using water, diiodomethane and formide as the reference liquids [3, 17]. In brief, the microalgal suspensions were harvested, washed twice and resuspended in deionized water. Algal lawns were prepared by filtering the suspensions on 0.45 μm nitrocellulose membrane until complete covering the membranes. The lawn was then placed in a Petri dish on 1% (w/v) agar layer containing 10% (v/v) glycerol and stored for 2 h to homogenize the moisture content. The filters were then dried in air at room temperature for 60 min in order to obtain relatively stable contact angles. Contact angles (at least 8 drops) were determined using a tensiometer (K100, Krüss GmbH, Hamburg, Germany).

#### Surface hydrophobicity parameters determinations

The results from the contact angle measurements with three solvents were used to calculate the values of algal surface free energy parameters using the following Eq (1) [18]:

$$\cos\theta =-1+\frac{2\sqrt{\gamma_{s}^{\mathrm{LW}}\gamma_{l}^{\mathrm{LW}}}}{\gamma_{l}}+\frac{2\sqrt{\gamma_{s}^{+}\gamma_{l}^{-}}}{\gamma_{l}}+\frac{2\sqrt{\gamma_{s}^{-}\gamma_{l}^{+}}}{\gamma_{l}}$$ (1)

Where *θ* is the contact angle and *γ*_l =_ *γ*^LW^+*γ*^AB^.

Where *γ*^LW^ accounts for the Lifshitz-van der Waals (LW) component of the surface free energy, and subscripts *s* and *w* respectively refer to the surface and water. *γ*^-^ and *γ*^+^ are the electron donor and acceptor parameters, respectively of the Lewis acid-base component *γ*^AB^ (*γ*^AB^ = $2\sqrt{\gamma^{+}\gamma^{-}}$), while subscripts *s* and *l* respectively refer to the surface and probe liquid. In Eq (2), contact angle of the apolar liquid, diiodomethane, was used to quantify the apolar surface energy component *γ*_s_^LW^, since *γ*_l_^-^ and *γ*_l_^+^ are both equal to zero. Moreover, the contact angles measured with the other two probe liquids, water and formamide, were used to solve for the other two unknown surface energy parameters, *γ*_s_^-^ and *γ*_s_^+^.

The degree of hydrophilicity and hydrophobicity of the algal surfaces, Δ*G*_coh_, can be calculated through the surface tension components of the interacting entities according to Eq (2)[19–21]:

$$\Delta G_{coh}=-2{\left(\sqrt{\gamma_{s}^{\mathrm{LW}}}-\sqrt{\gamma_{w}^{\mathrm{LW}}}\right)}^{2}+4(\sqrt{\gamma_{s}^{+}\gamma_{w}^{-}}+\sqrt{\gamma_{s}^{-}\gamma_{w}^{+}}-\sqrt{\gamma_{s}^{+}\gamma_{s}^{-}}-\sqrt{\gamma_{w}^{+}\gamma_{w}^{-}})$$ (2)

In this approach, a negative Δ*G*_coh_ indicates hydrophobicity where surface-surface interactions are stronger than surface-water interactions. Alternatively, a positive one indicates hydrophilicity [22].

#### Potentiometric titrations

Potentiometric titrations were performed in order to determine the deprotonation constants and concentrations of the specific functional groups present on the algal cell walls[8]. In detail, 50 mL algae samples were taken by centrifugation (5000 ×*g*, 10 min) and then washed twice with 0.001M Na_2_EDTA to remove adsorbed salts. The cells were subsequently washed three times with 0.1 M NaNO_3_ and the volume brought up to 45 mL with 0.1 M NaNO_3_. The potentiometric measurements were carried out at 25±0.5°C using a pH meter (Sartorius, German) under a nitrogen stream to avoid dissolution of CO_2_ in the solution. Suspension pH was firstly lowered to 3.0 using 0.1 M HCl and then titrated up to pH of 10 using 0.1 M NaOH. 20 μL NaOH was added each time and the pH recorded. Raw data from titrations were analyzed using Profit 2.1 based on the Donnan Shell Model [10].

### Statistical analysis

Results are averages of triplicates and the values in each graph and table are shown with 5% error bars. Analysis of *t*-test and Pearson’s Correlation was performed using SPSS 18.0 package (SPSS, Chicago, IL), with values of 0.05 selected for significance.

## Results and discussion

### Changes in algal lipid content with changing growth phase

**Fig 1:**
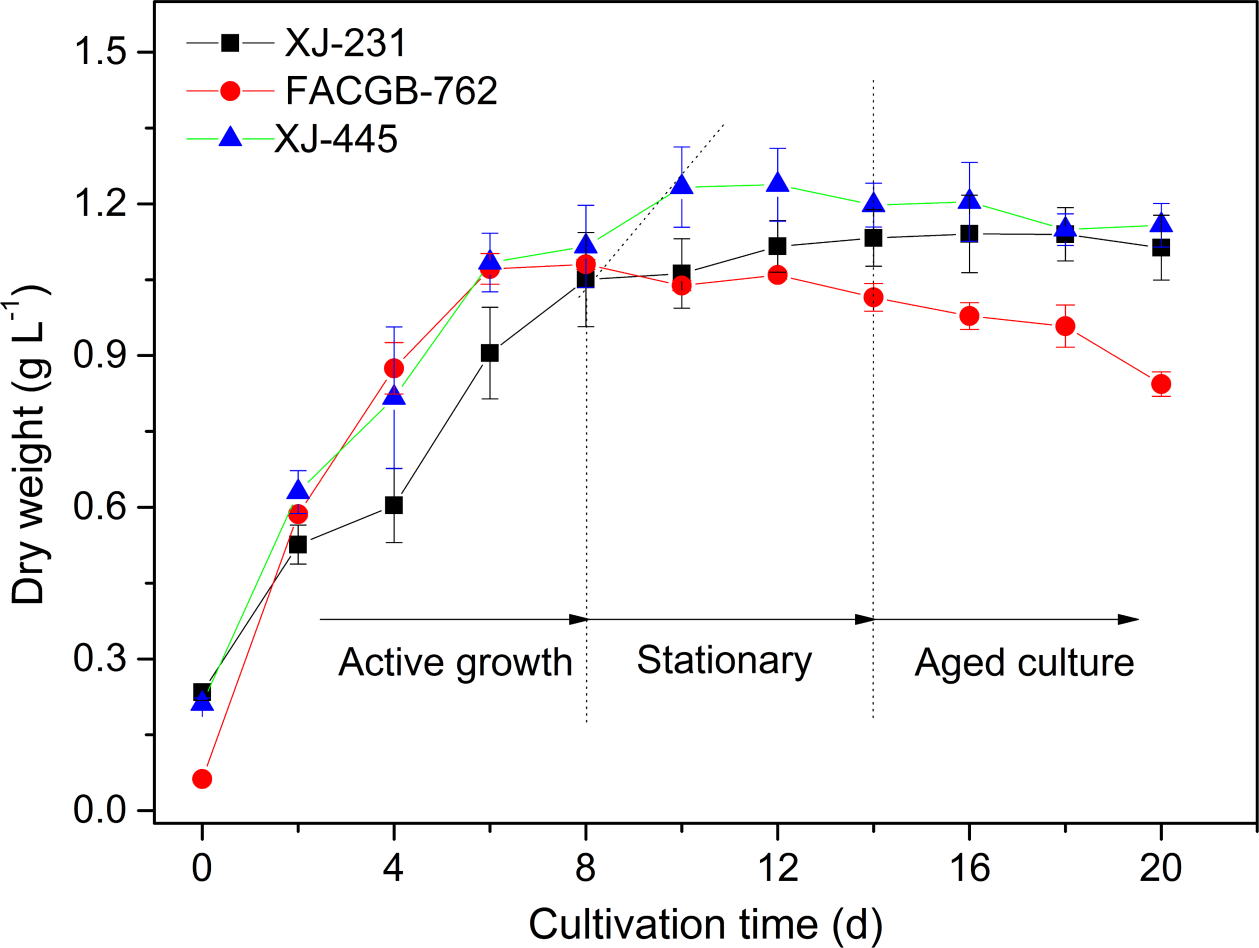
Changes in dry weight during 20-day cultivation of *Botryococcus* sp. FACGB-762, *Chlorella sp*. XJ-445 and *D*. *bijugatus* XJ-231.

Changes in the biomass concentration of *Botryococcus* sp. FACGB-762, *Chlorella* sp. XJ-445 and *D*. *bijugatus* XJ-231 were shown in Fig 1. A nearly linear increase in cell growth was observed in the first 6 days for *Botryococcus* sp. FACGB-762, in the first 8 days for both *Chlorella* sp. XJ-445 and *D*. *bijugatus* XJ-231, which was regarded as the active growth phase. It was observed that *Botryococcus* showed the highest growth rate in terms of slope at active growth phase (Fig 1). Generally, the growth rate of *Botryococcus* was lower than *Chlorella* and *Desmodesmus*., however, showed the highest in this study. It might be because that experimental setup in this study was more favorable for *Botryococcus* [23, 24] since *Chlorella* and *Desmodesmus* favored the growth conditions like higher temperature and higher light intensity [25–27]. After active growth phase, the growth of the three microalgae leveled off, and declined after 14 days cultivation. Therefore, samples from day 4, day 12 and day 20 of the three algal cultures, defined as active growth phase, stationary growth phase and aged culture phase respectively, were taken and used for lipid determination and subsequent cellular surface property analysis.

**Fig 2:**
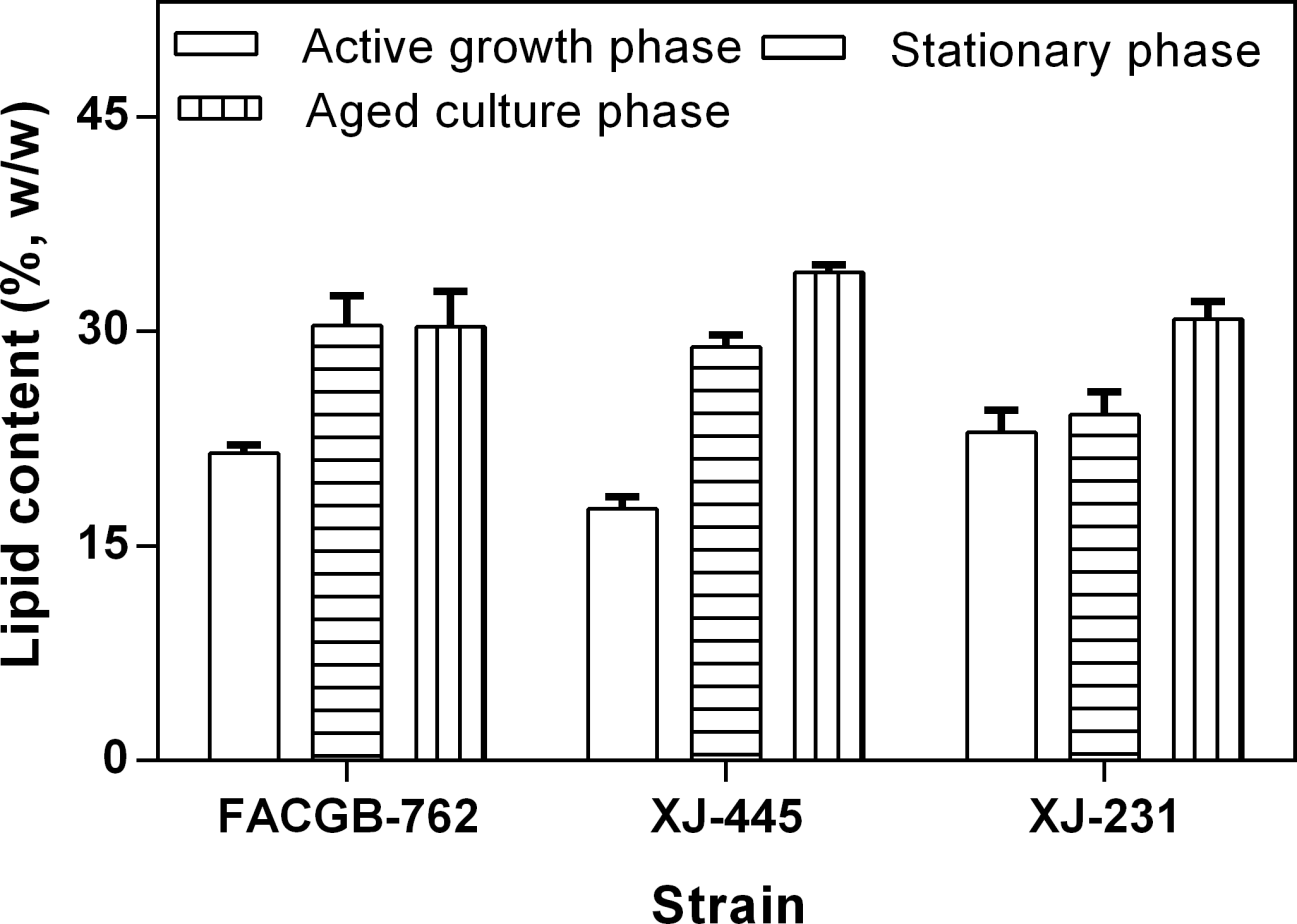
Lipid contents of *f Botryococcus sp*. FACGB-762, *Chlorella sp*. XJ-445 *and D*. *bijugatus* XJ-231 under different growth phases.

Lipid content of the three microalgae from different growth phases was shown in Fig 2. The lipid content increased significantly with the growth stages (*P<0*.*05*) for *Chlorella* sp. XJ-445 as expected. Though significantly enhanced in lipid content when comparing with active growth and aged culture phases, there is no significant difference in lipid content between stationary and aged culture phase for *Botryococcus* sp. FACGB-762, (*P>0*.*05*), and between active growth and stationary phase for XJ. 231 (*P>0*.*05*). For *Chlorella* sp. XJ-445, the lipid content increased 2-fold, from active growth phase (17.6%) to aged culture phase (34.1%). The observations were consistent with many reports in which an increase in lipid content with the growth phases was observed due to the depletion of nutrients [28].

### Changes in algal cell characteristics with changing growth phase

#### Zeta potential and particle size

**Fig 3:**
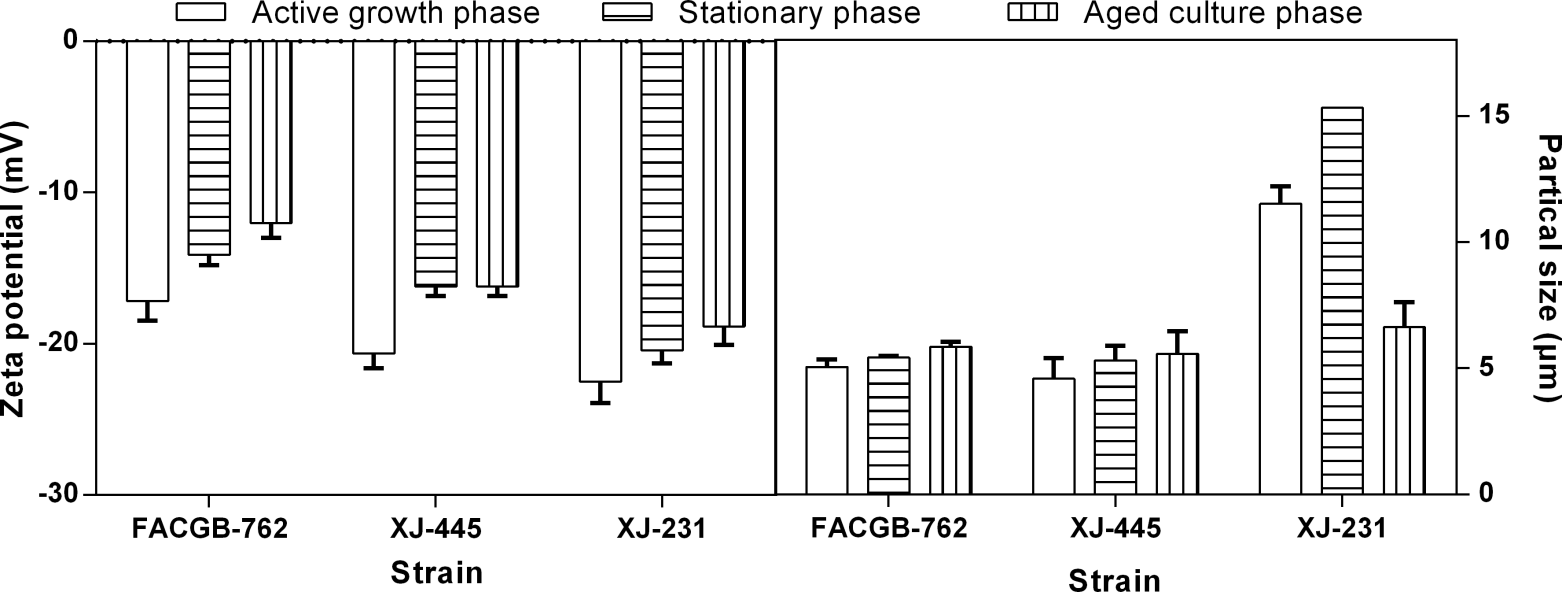
Zeta potential (A) and sample size of *Botryococcus sp*. FACGB-762, *Chlorella sp*. XJ-445 *and D*. *bijugatus* XJ-231 under different growth phases. The *d50* (particle size at 50% cumulative undersize) termed sample size was reported.

To investigate the influence of growth phase on the surface charge of microalgae, cells of *Botryococcus* sp. FACGB-762, *Chlorella* sp. XJ-445 and *D*. *bijugatus* XJ-231 from different growth phases were obtained for zeta potential measurement. Fig 3A showed a significant decrease in the net electronegative zeta potential for all three microalgal species from active growth phase to stationary phase (*P<0*.*05*). The observation was consistent with the previous study by Danquah et al. (2009). As for the aged culture phase, the net zeta potential showed different changes in the different microalgae. For *Botryococcus* sp. FACGB-762, zeta potential continued to decrease, while it leveled off in *Chlorella* sp. XJ-445, and increased in *D*. *bijugatus* XJ-231 cultures. The cellular size showed the opposite to the net zeta potential pattern (Fig 3B, Table 1).

**Table 1:** Sample size expressed as *d50*, contact angles and surface physicochemical properties determined for *Botryococcus sp*. FACGB-762, *Chlorella sp*. XJ-445 and *D*. *bijugatus* XJ-231.

Stain		*Botryococcus* sp. FACGB-762			*Chlorella* sp. XJ-445			*D*. *bijugatus*. XJ-231		
Growth phase		active growth	stationary	aged culture	active growth	stationary	aged culture	active growth	stationary	aged culture
Sample size (*d50*, μm)		5.05±0.29	5.42±0.06	5.84±0.21	4.58±0.83	5.30±0.59	5.57±0.09	11.52±0.69	15.33±0.06	6.62±0.99
Contact angles (°)	*θ*_W_	28.7±1.9	57.3±8.1	75.3±3.6	21.6±1.4	33.8±1.4	21.7±1.8	34.8±5.2	38.8±3.5	30.1±2.8
	*θ*_F_	34.4±7.1	53.1±4.4	70.9±2.1	24.1±2.8	19.5±0.7	10.6±2.3	51.1±4.3	63.3±4.1	38.6±11.1
	*θ*_D_	35.2±5.2	44.5±2.0	46.9±3.4	42.5±6.8	45.4±2.5	36.9±2.0	37.5±5.9	51.8±2.3	48.5±2.9
Surface tension parameters and free energy of hydrophobic interaction (mJ m^-2^)	*γ*_s_^LW^	41.9±2.5	37.3±1.1	36.0±1.8	38.3±3.4	36.8±1.3	41.1±1.0	40.7±2.9	33.3±1.3	35.1±1.6
	*γ*_s_^AB^	3.1±2.2	2.5±0.6	0	11.0±4.7	15.3±1.8	14.3±1.4	0	0	10.4±3.6
	*γ*_s_^-^	55.2±7.1	31.1±13.5	20.9±16.5	55.0±7.0	51.3±0.5	48.2±0.8	64.3±2.7	74.8±1.2	57.5±13.2
	*γ*_s_^+^	0	0.2±0.2	0	0	2.3±1.8	1.1±0.2	0	0	0.6±0.5
	*ΔG*_coh_	39.4±11.4	16.4±9.9	-38.1±13.2	36.2±11.6	29.2±0.9	24.3±0.8	54.0±2.6	70.2±1.4	40.9±17.9

Values are presented as the mean ± standard deviation of two independent experiments. *θ*_W_, contact angle with water; *θ*_F_, contact angle with formamide; *θ*_D_, contact angle with diiodomehane; *γ*_s_^LW^, Lifshitz-van der Waals component of the surface free energy; *γ*_s_^AB^, Lewis acid-base component of the surface free energy; *γ*_s_^-^, electron donor component; *γ*_s_^+^, electron acceptor component; *ΔG*_coh_, free energy of hydrophobic interaction.

**Fig 4:**
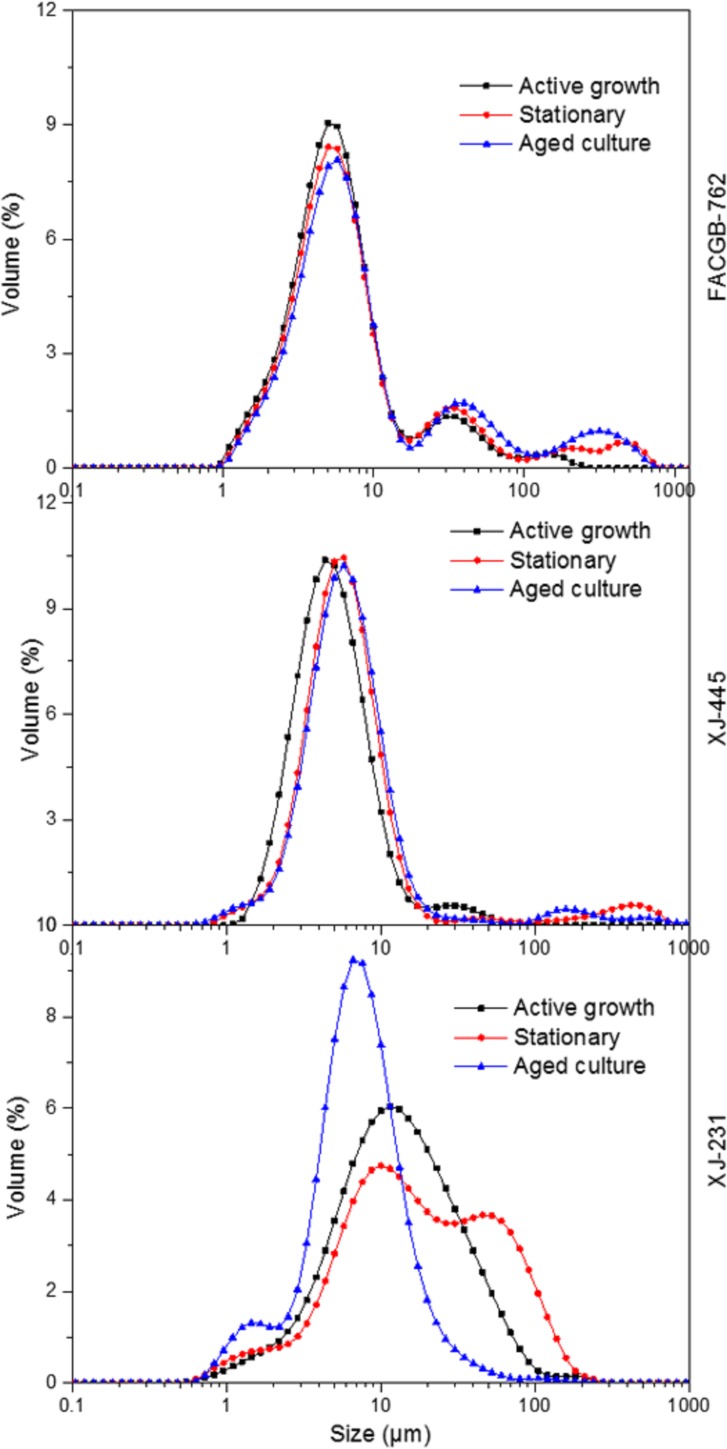
Sample size distribution of *Botryococcus sp*. FACGB-762, *Chlorella sp*. XJ-445 *and D*. *bijugatus* XJ-231 at different growth phase.

During the active growth phase, due to the high rate of cell growth and unicellular mobility, there was rare intercellular interaction between individual cells, which caused a highly net electronegative zeta shield around the cells, creating a massive repulsion between the cells [11]. However, when stepping to the late growth phases, the metabolism rate of the microalgal cells was low and the unicellular mobility was reduced [11]. This caused a less electronegative zeta shied around the individual cells, which could be observed in *Botryococcus* sp. FACGB-762 and *Chlorella* sp. XJ-445. As for the different observation in *D*. *bijugatus* XJ-231, the decreased particle size during aged culture phase was largely attributed to the 4-cell group dispersing to single cell as presented in Fig 4. This also generated a reduced electronegative zeta shied around the individual cells. Therefore, for single cell, it was implied that the net zeta potential decreased and particle size increased with growth phase for all the three microalgae.

#### Hydrophobicity

The surface tension parameters and free energy of hydrophobic interaction determined using the approach of van Oss et al. (2008) [18] for *Botryococcus* sp. FACGB-762, *Chlorella* sp. XJ-445 and *D*. *bijugatus* XJ-231 at different growth stages were shown in Table 1. Higher contact angles were measured for microorganisms presenting more hydrophobic surfaces (lower *ΔG*_coh_ values). For all the strains, the degree of hydrophobicity was higher in active growth phase than that in aged culture phase in terms of *ΔG*_coh_ values. The lowest average *ΔG*_coh_ value was found in aged culture phase, although for *D*. *bijugatus* XJ-231, the values of *ΔG*_coh_ from active growth and aged culture phase showed no significant differences *(P>0*.*05)*. For *D*. *bijugatus* XJ-231, the higher *ΔG*_coh_ found at stable phase might be caused by the fact that higher cell size of 4-cell pattern made a rougher surface, which led a higher lower contact angle measurement and a higher *ΔG*_coh_ calculation. The degree of hydrophobicity was associated with the extent of attachment of cells to a surface, and cells with higher degree of hydrophobicity would prefer to stick on the surface of the bioreactor.

In addition, the results showed that the electron donor and acceptor parameters of the algal surfaces ranged from 20.9±16.5 to 74.8±1.2 mJ m^-2^ and from 0 to 2.3±1.8 mJ m^-2^ for the three strains. For a specific microorganism, *γ*_s_^-^ and *γ*_s_^+^ values were not statistically different at the different growth phases (*P>0*.*05*). Higher values determined for *γ*_s_^-^ indicate that the studied microalgae showed a strong electron-donating property. It should be stated that similar results were obtained in previous studies by Ozkan and Berberoglu (2013) and Gonçalves et al. (2015) [3, 29]. In addition, *γ*_s_^+^ values close to zero are in accordance with the Oss et al. (2008) model, which predicts almost the non-existence of electron acceptor parameters [3].

#### Surface functional groups

The titration experiments were used to determine the effect of growth phase on the site concentrations of ionizable functional groups on the algal surface of the three organisms. The results from the Protofit program based on Donnan Shell Model simulation were summarized in Table 2. For all species at all stages, site densities were expressed as 10^−3^ moles of sites per gram of cell, which were consistent with the previous studies for microalgae [8, 16]. According to Hadjoudja et al. (2010), deprotonation constants of major functional groups were as follows: carboxyl (pKa = 2–6), phosphoryl (pKa = 5.6–7.2) and amine (pKa = 8.6–9.0) and hydroxyl (pKa = 8–12). Thus, the pK1, pK2 and pK3 for all the three strains can be assigned to carboxyl, phosphoryl and hydroxyl functional groups, respectively. It was found that microalgae had different deprotonation constants and different site concentrations of functional group at different growth phase. For *Botryococcus* sp. FACGB-762 and *D*. *bijugatus* XJ-231, carboxyl functional groups only occurred at stable phase, while *Chlorella* sp. XJ-445 had carboxyl functional groups at all three growth phases. This may be due to the limitation of the testing methods and further work is needed to focus on the method development in the microalgal cell surface characterization.

**Table 2:** Potentiometric titration modeling results for *Botryococcus sp*. FACGB-762, *Chlorella sp*. XJ-445 *and D*. *bijugatus* XJ-231 under different growth phases.

Stain	Growth phase	pK1	C_*1*_(g mol^-1^)	pK2	C_*2*_ (g mol^-1^)	pK3	C_*3*_ (g mol^-1^)	Ctot
*Botryococcus* sp. FACGB-762	active growth			7.29±0.13	0.09±0.02	10.40±0.22	0.27±0.06	0.36±0.14
	stationary	6.17±0.15	0.13±0.04	6.71±1.25	0.09±0.01	9.71±0.32	0.37±0.16	0.71±0.04
	aged culture			6.56±0.43	0.53±0.09	10.60±0.35	2.15±0.35	2.68±1.53
*Chlorella* sp. XJ-445	active growth	4.85±0.57	0.06±0	8.22±0.62	0.06±0.03	10.82±2.63.	0.11±0.05	0.40±0.10
	stationary	6.30±0.33	0.15±0.07	6.71±0.58	0.06±0.03	10.03±0.15	0.96±0.08	1.25±0.16
	aged culture	6.52±0.40	0.27±0.22	7.04±0.05	0.09±0.09	10.55±0.29	3.24±0.94	3.57±2.88
*D*. *bijugatus* XJ-231	active growth			6.41±0.09	0.19±0.03	9.87±0.46	0.47±0.04	0.52±0.31
	stationary	5.74±0.06	0.15±0.01	6.85±0.08	0.17±0.09	9.96±0.46	0.78±0.03	1.19±0.14
	aged culture			6.32±0.03	0.45±0.06	9.97±0.13	2.95±0.62	3.49±0.45

*C* donates the concentration of the corresponding deprotonation constants of functional groups (pK value); Ctot is the total concentration of all the functional groups determined.

It was noted that all the strains exhibited predominantly hydroxyl sites and the hydroxyl sites concentration increased with the growth phase from active growth to aged culture phase. Because of the high concentration of hydroxyl sites, the total concentration of surface functional group also increased with the growth stage. In general, the amount of dissolved organic matter (DOM) in the medium excreted from algal cells increased with the growth stage [10, 30]. The increase in the total concentration of functional groups might also be due to the DOM formation and adhering to the cellular surface. Furthermore, algal biomass from late growth phase with higher concentration of functional groups can be used in heavy metal removal, in which the abundant carboxyl and hydroxyl functional groups can react with the heavy metals [31].

### Interaction of lipid content and surface properties

To further explore the relationship between the lipid content and surface properties, a test of Pearson’s correlation was conducted. The results were listed in the Table 3. As presented, most surface properties including the zeta potential, sample size or surface tension parameters had no significant relationship with the lipid content. However, as for the functional groups, it was interesting to find that the lipid content had a significantly positive correlation with the concentration of hydroxyl functional groups even in the different algal strains at different growth phase. In addition, the lipid content was positively correlated to the concentration of phosphoryl functional groups and also positively with the total concentration of functional groups with significance values of 0.067 and 0.063 (Table 3). The possible reason is that the lipid synthesis in the microalgal cells at different growth phase may cause some changes in the cell membrane and further influence the constituents of cell wall, which bring the changes in the concentration of the functional groups. Also, the changes in the lipid content at different growth phase were largely associated with the gradually depletion of the nutrients in the medium, thus further work is needed to focus on effect of cultivation conditions on the algal surface properties.

**Table 3:** Results of Pearson’s correlation between lipid content and the surface properties.

	Zeta potential	Sample size	*γ*^*LW*^	*γ*^*+*^	*γ*^*-*^	*γ*^*AB*^	*γ*_*s*_	*ΔG*_*coh*_	*C*_*2*_	*C*_*3*_	*C*_*tot*_
Pearson correlation with lipid content	0.203	-0.027	-0.246	0.328	0.002	0.462	0.353	-0.028	0.545	0.699*	0.551
*Significance*	0.526	0.933	0.441	0.298	0.994	0.131	0.26	0.932	0.067	0.039	0.063

*γ*^LW^, Lifshitz-van der Waals component of the surface free energy; *γ*^AB^, Lewis acid-base component of the surface free energy; *γ*^-^, electron donor component; *γ*^+^, electron acceptor component; *γ*_*s*_, surface free energy; *C*_*2*_ donates concentration of phoshoryl functional groups, while *C*_*3*_ represents the concentration of hydroxyl functional groups. 0.05 was selected as the significant level.

## Conclusions

Throughout the growth phases, the lipid content of microalgae increased. While for the surface properties, the parameters such as particle size, the degree of hydrophobicity, and the total concentration of functional groups increased with decreasing net surface zeta potential. It was suggested that the growth phase had significant influences not only on the lipid content but also on the surface characteristics. Moreover, the lipid content significantly positively related to the concentration of hydroxyl functional groups. This study helps to fill the gap of cellular surface properties characterization during the growth periods in the field of using oleaginous microalgae for biofuel production.
